# SPECT/CT for Lymphatic Mapping of Sentinel Nodes in Early Squamous Cell Carcinoma of the Oral Cavity and Oropharynx

**DOI:** 10.1155/2011/106068

**Published:** 2010-09-06

**Authors:** Haerle Stephan K., Stoeckli Sandro J.

**Affiliations:** ^1^Department of Otolaryngology-Head and Neck Surgery, University Hospital Zurich, Frauenklinikstrasse 24, 8091 Zurich, Switzerland; ^2^Department of Otolaryngology-Head and Neck Surgery, Kantonsspital St. Gallen, Rorschacherstra*β*e 95, 9000 St. Gallen, Switzerland

## Abstract

Adequate staging and treatment of the neck in squamous cell carcinoma of the oral cavity and oropharynx (OSCC) is of paramount importance. Elective neck dissection (END) of the clinical N0-neck is widely advocated as neck treatment. With regard to the prevalence of 20–40% of occult neck metastases found in the ND specimens, the majority of patients undergo surgery of the lymphatic drainage basin without therapeutic benefit. Sentinel node biopsy (SNB) has been shown to be a safe, reliable and accurate alternative treatment modality for selected patients. Using this technique, lymphatic mapping is crucial. Previous reports suggested a benefit of single photon emission computed tomography with CT (SPECT/CT) over dynamic planar lymphoscintigraphy (LS) alone. SPECT/CT allows the surgeon for better topographical orientation and delineation of sentinel lymph nodes (SLN's) against surrounding structures. Additionally, SPECT/CT has the potential to detect more SLN's which might harbour occult disease, than LS. SPECT/CT is recommended to be used routinely, although SPECT/CT is not indispensable for successful SNB.

## 1. Background

Squamous cell carcinoma of the oral cavity and oropharynx (OSCC) accounts for one of the most common cancers worldwide, with more than a quarter million new cases annually [1]. The presence or absence of lymph node involvement is of paramount importance for prognosis and therapy decision [2, 3]. Therefore, an adequate staging and management of the neck is needed. The most challenging issue remains the treatment of the clinically and radiologically negative neck. Most centers throughout the world advocate elective neck dissection (END) for histopathologic staging and removal of microscopic disease in this situation. With regard to the prevalence of 20%–40% of occult neck metastases found in the neck dissection specimens, the majority of patients undergo surgery of the lymphatic drainage basin without therapeutic benefit. Sentinel node biopsy (SNB) has been shown to be very accurate in selecting patients who benefit from elective neck treatment and sparing the costs and morbidity to the others. Detection of the sentinel nodes by lymphatic mapping is crucial with this technique. Single-photon emission computed tomography with CT (SPECT/CT) has been recently introduced to enhance the diagnostic accuracy of preoperative lymphoscintigraphy.

## 2. Sentinel Node Biopsy

By definition, the sentinel lymph node (SLN) is the first draining lymph node to receive lymphatic drainage from a primary tumor of a specific site [4]. In case of lymphatic spread, the lymphatic drain will first pass the SLN. All following nodes may be reached only subsequently by the disease. Therefore, selective excision of the SLN with subsequent thorough histopathologic work-up reflects adequately the nodal status of the remaining neck [5]. Since Alex and Krag [6] have described their first experience with SNB for OSCC, the technique has gained large popularity and many centers followed with validation and observational studies [7–9]. Lymphatic mapping of the SLN in the complex head and neck area has been shown to be essential [10]. The problems in the head and neck area are threefold: first, there is a high density of lymph nodes, second, the structure of these nodes shows an unique complexity of lymphatic pathways, and third, the SLNs are located in close proximity to the primary tumor. Therefore, sophisticated lymphatic mapping techniques are required. During the preoperative setting, a dynamic lymphoscintigraphy (LS) assesses the individual draining pattern after injection of radiolabeled particles around the primary tumor. The intraoperative use of a hand-held gamma probe helps the surgeon to localise and excise the first echelon lymph nodes. The success of this technique has been abundantly reported in the literature and well-documented guidelines do exist [11]. As with breast cancer, preliminary reports showed a new imaging technology with promising results: SPECT/CT.

## 3. Patient's Selection

For SNB of the OSCC, patients with stages I and II (T1 and T2) disease and no clinical and radiological evidence of cervical lymph node involvement are eligible. Absence of suspicious or metastatic lymph nodes is based on palpation, ultrasound with fine needle aspiration cytology (FNAC), or contrast-enhanced computed tomography (CT), or magnetic resonance imaging (MRI), or ^18^F-fluoro-2-deoxy-D-glucose positron emission tomography (^18^F-FDG-PET)/CT. With regard to conventional imaging, lymph nodes greater than 1.5 cm in level II and greater than 1 cm in all other levels, or lymph nodes with round shape, central necrosis, and peripheral contrast enhancement are considered pathologic. In metabolic imaging, a lymph node with a clearly higher FDG-uptake compared to the background and anatomically corresponding to a lymph node in the low-dose CT scan is considered pathologic.

## 4. Tracer

To assess the individual lymphatic drainage pattern, a peritumoral injection of radiolabeled particles is performed. The particles will enter the lymphatic capillaries and accumulate in the first draining node. There is a variety of colloidal and soluble tracers available although most trials report using Tc-99m-labeled human serum albumin colloid (Nanocoll, GE Healthcare). With its particle size of 8–30 nm, Nanocoll migrates to the sentinel node within minutes and remains there until the next day [11, 12]. This allows a flexible way for planning surgery to take place.

## 5. Imaging

A standard technique for preoperative imaging is the use of a gamma camera for lymphoscintigraphy to assess the individual drainage pattern of the injected radiolabeled tracer via the capillaries to the larger collector lymphatics [13]. Imaging will be performed either the day before or the day of surgery. According to *the joint practice guidelines for radionuclide lymphoscintigraphy*, the setting of the camera is proposed to be as follows. A large-field-of-view gamma camera provided with a high- or ultrahigh-resolution low-energy collimator should be used, with a 10%–20% window centered on the 140-keV energy peak of Tc-99m [11]. The gamma camera should be routinely checked for quality control as proposed in published protocols [14]. 

Immediately after the injection of the radiotracer, the lymphatic drainage is monitored dynamically with the gamma camera in the anteroposterior projection (1 image/3 minutes). The lymphatic drainage is then observed by the nuclear medicine specialist and the HN-surgeon at the monitor. When accumulation of the radiotracer in the first echelon node(s) occurs, the dynamic imaging can be interrupted and static imaging in the anterior-posterior, lateral and, if necessary, anterior oblique view can be performed. For the different projections, a three-headed camera is recommended. To be able to localize the nodes in a three-dimensional view, static images in at least two projections are needed. The patient is imaged in the supine position with head up [11]. 

Most reports in the literature use the term sentinel node interchangeably for lymphoscintigraphy and SNB. As most tracer accumulations or *hot spots* detected by lymphoscintigraphy or SPECT/CT correspond to more than one ultimately excised sentinel lymph node, the term *hot spot* should be used in the context of planar imaging or fused imaging whereas *sentinel lymph node* should be used in the context of surgical SNB. 

Intraoperatively, the surgeon will be guided to the sentinel nodes by a hand-held gamma probe containing a radiation detector with surrounding metal shielding and a collimated tip. The response related to the detected count rate is provided by a connected analyzer [11].

Using this technique, SNB has become a safe and reliable method to detect SLNs with a previously published SLN detection rate of 96% [5].

## 6. SPECT/CT for Sentinel Node Mapping in HNSCC: A Comparison in the Literature

**Figure 1:**
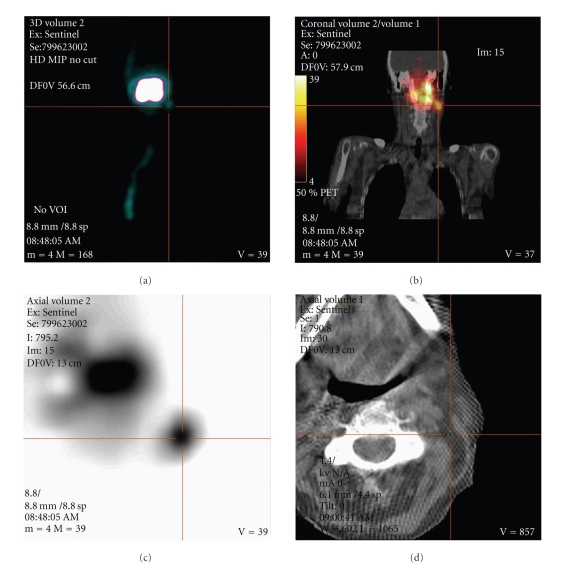
64-year-old female suffering from a left-sided tongue cancer. (a) shows the MIP (maximum intensity projection)—image of SPECT acquisition in anteroposterior view. A large uptake is seen at the injection site with a small, focal uptake of the left lower boarder (cross hair). (b) shows a fused coronal SPECT/CT image that localises the small focal uptake in the neck region level IIA/B. (c) shows the axial SPECT image with the corresponding cross hair in the sentinel node with a clear delineation from the injection site. (d) shows the corresponding low-dose CT scan localising the uptake by linked cross hair into the neck level IIA/B.

Besides the previously described imaging technique using a preoperative lymphoscintigraphy, novel systems composed of a gamma camera and a CT scan combined in the same device have been recently introduced into clinical practice. Single photon emission CT (SPECT) and CT data are acquired at the same clinical setting without changing the patient's positioning, thus allowing for generation of accurate fused images combining the functional data of SPECT with the anatomical data of the CT scan (Figure 1). Different centers have already reported on their experience with SPECT/CT for SLN mapping in early OSCC, however, with contradictory results [11, 15–24]. In 2000, Even-Sapir et al. described the fusion of the SPECT lymphoscintigraphy data with CT using a hybrid gamma-camera and a low-dose CT system that allows SPECT and CT to be performed at the same time without changing the patient's position [25]. Three years later, the same author introduced the hybrid SPECT/CT system into sentinel node mapping of HNSCC [15]. In 2004, two feasibility studies using planar lymphoscintigraphy and SPECT/CT were published [16, 17]. Lopez et al. included ten patients stating that they believe that SPECT/CT will become a useful tool for sentinel node mapping [16]. Wagner et al. found an additional value by using SPECT/CT for sentinel node mapping than lymphoscintigraphy alone [17]: they have found an additional lymph node nearby the submandibular gland which has only been detected by SPECT/CT. This lymph node has been overlooked by planar lymphoscintigraphy and the intraoperative gamma probe as the radioactive scattering from the primary obscured the location of the radiolabeled SLNs. The same problem has been shown by other authors in the earlier period of radioguided imaging [26, 27] and was thought be resolved by introducing SPECT/CT. Thomsen et al. found SLNs close to the primary difficult to detect. Therefore, added oblique planar images and/or tomographic scans would help to overcome this problem [18]. Terada et al. also performed a feasible study on SPECT/CT and HN mucosal carcinoma and concluded that they were able to extract all the SLNs based on the fusioned images and to confirm its radioactivity with the gamma probe without the adverse effect of overlapping radioactivity from the primary site [19]. Khafif et al. included 22 patients with biopsy proven OSCC and found an improved identification of the SLNs of 30% compared to planar imaging [20]. Bilde et al. included 34 consecutive patients with stages I and II OSCC undergoing planar lymphoscintigraphy and SPECT/CT. After all, SPECT/CT demonstrated an extra SLN in 47% compared to lymphoscintigraphy alone [21]. In the same year, Keski-Säntti et al. were the first and only authors who found no additionally revealed SLNs by SPECT/CT compared to planar imaging [22]. They concluded that despite the better topographical orientation achieved by SPECT/CT, it is not necessarily needed for preoperative lymphatic mapping. The largest single institutional cohort study was done by Haerle et al. A total of 58 patients undergoing SNB with preoperative lymphoscintigraphy and SPECT/CT have been described. Lymphoscintigraphy showed full concordance with SPECT/CT in 81% of the cases. SPECT/CT was able to detect additional HS in eleven patients, in one case even with additional metastatic disease. Therefore, in conclusion, SPECT/CT has the potential to detect more SLNs, which might harbour occult disease, than Lymphoscintigraphy alone. However, with regard to the excellent results achieved with LS and the intraoperative use of the gamma probe, SPECT/CT is not indispensable for successful SNB. The additional hot spots have all been detected in the same levels or in levels close to those in which lymphoscintigraphy has already shown hot spots. Therefore, both imaging modalities have difficulties in detecting level I sentinel nodes close to the injection site [23]. In summary, the reported series looking at preoperative sentinel node mapping for OSCC were all smaller series apart from the latter series [23]. An overview of the different series is shown in Table 1.

## 7. In the Future

Wherever applicable and affordable, SPECT/CT might become a routine preoperative imaging solution in the context of SNB for OSCC. As technical developments in SPECT/CT are ongoing [28] and high-resolution multislice CT scanners and the use of intravenous contrast will be integrated in SPECT/CT systems, even further improved spatial resolution of CT images and a better delineation of tumor adjacent structures are available. Together with the use of novel portable gamma-camera systems tested recently [29], possibly the future will provide an integrated system that combines fused imaging with an intraoperative hand-held gamma-camera enabling three-dimensional visualization [30]. Apart from imaging development, new tracers may be integrated in the process of more accurate detection of the sentinel lymph nodes.

## 8. Conclusions

The technique of SNB offers a reliable, safe, and individual treatment plan for each patient's unique lymphatic drainage pattern causing low morbidity. The use of this successful technique allows the surgeon to select patients for elective neck dissection of the N0-neck. The use of preoperative planar lymphoscintigraphy and an intraoperative hand-held gamma probe results in excellent sentinel node-detection rates. SPECT/CT allows the surgeon for better topographical orientation and delineation of SLNs against surrounding structures, for example, muscles, vessels, and bones. Additionally, the surgical time may be reduced with regard to better spatial resolution. SPECT/CT has the potential to detect more SLNs, which might harbour occult disease, than lymphoscintigraphy alone. Therefore, we recommend using SPECT/CT routinely, although SPECT/CT is not indispensable for successful SNB.

## Figures and Tables

**Table 1:** An overview of various studies using SPECT/CT in the context of lymphatic mapping for SNB in OSCC.

Study group	Number of patients (n)	SPECT/CT and the reported detection of SLN's	The value of SPECT/CT according to the authors
Even-Sapir et al. [15]	6	3 additional nodes detected in 6 patients compared to lymphoscintigraphy alone	SPECT/CT adds data that is of clinical relevance to SNB in patients with mucosal HNSCC

Lopez et al. [16]	10	100% visualization of the SLN's by SPECT/CT	SPECT/CT is shown to be an effective method for anatomic localization of the SLN's in N0 OSCC

Wagner et al. [17]	30	11 additional nodes out of 49 SLNs detected compared to lymphoscintigraphy alone	SPECT/CT adds additional information regarding nodes that are adjacent to the primary lesion

Thomsen et al. [18]	40	SPECT/CT and/or added oblique images revealed extra nodes in 15/40 patients.	SPECT/CT has added information which could not have been obtained from planar lymphoscintigraphy

Terada et al. [19]	15	100% visualization of the SLN's by SPECT/CT	SPECT/CT proved to be an easy, accurate, and reliable method

Khafif et al. [20]	20	SPECT/CT improved SLN identification and/or localization compared with planar images in 6 patients (30%)	SPECT/CT provides additional preoperative data of clinical relevance to SNB in patients with OSCC

Bilde et al. [21]	34	SPECT/CT demonstrated extra SLN's compared to planar imaging in 15 out of 32 patients (47%)	SPECT/CT detects more SLN's than lymphoscintigraphy and provides additional anatomical and spatial information about their localization.

Keski-Säntti et al. [22]	15	1 additional SLN located in the jugular chain detected compared to lymphoscintigraphy alone	SPECT/CT enables more accurate localization of the SLN's, but it rarely reveals SLN's, that are not detected on planar images.

Haerle et al. [23]	58	11 additional hot spots could be revealed by SPECT/CT compared to lymphoscintigraphy alone. In one case even with additional occult disease.	SPECT/CT has the potential to detect more SLN's, which might harbour occult disease, than lymphoscintigraphy alone.

SNB: sentinel node biopsy; OSCC: oral/oropharyngeal squamous cell carcinoma; HNSCC: head and neck squamous cell carcinoma; SLN: sentinel lymph node.
